# SMCHD1 activates the expression of genes required for the expansion of human myoblasts

**DOI:** 10.1093/nar/gkae600

**Published:** 2024-07-12

**Authors:** Matthew Man-Kin Wong, Sarah Hachmer, Ed Gardner, Valeria Runfola, Eric Arezza, Lynn A Megeney, Charles P Emerson, Davide Gabellini, F Jeffrey Dilworth

**Affiliations:** Sprott Center for Stem Cell Research, Regenerative Medicine Program, Ottawa Hospital Research Institute; Ottawa, ON K1H 8L6, Canada; Department of Cellular and Molecular Medicine, University of Ottawa; Ottawa, ON K1H 8L6, Canada; Department of Cell and Regenerative Biology, University of Wisconsin; Madison, WI 53705, USA; Sprott Center for Stem Cell Research, Regenerative Medicine Program, Ottawa Hospital Research Institute; Ottawa, ON K1H 8L6, Canada; Department of Cellular and Molecular Medicine, University of Ottawa; Ottawa, ON K1H 8L6, Canada; Division of Genetics and Cell Biology, IRCCS San Raffaele Scientific Institute, Milano 20132, Italy; Sprott Center for Stem Cell Research, Regenerative Medicine Program, Ottawa Hospital Research Institute; Ottawa, ON K1H 8L6, Canada; Sprott Center for Stem Cell Research, Regenerative Medicine Program, Ottawa Hospital Research Institute; Ottawa, ON K1H 8L6, Canada; Department of Cellular and Molecular Medicine, University of Ottawa; Ottawa, ON K1H 8L6, Canada; Wellstone Muscular Dystrophy Program, Department of Neurology, University of Massachusetts Chan Medical School, Worcester, MA 01655, USA; Division of Genetics and Cell Biology, IRCCS San Raffaele Scientific Institute, Milano 20132, Italy; Sprott Center for Stem Cell Research, Regenerative Medicine Program, Ottawa Hospital Research Institute; Ottawa, ON K1H 8L6, Canada; Department of Cellular and Molecular Medicine, University of Ottawa; Ottawa, ON K1H 8L6, Canada; Department of Cell and Regenerative Biology, University of Wisconsin; Madison, WI 53705, USA

## Abstract

SMCHD1 is an epigenetic regulatory protein known to modulate the targeted repression of large chromatin domains. Diminished SMCHD1 function in muscle fibers causes Facioscapulohumeral Muscular Dystrophy (FSHD2) through derepression of the *D4Z4* chromatin domain, an event which permits the aberrant expression of the disease-causing gene *DUX4*. Given that SMCHD1 plays a broader role in establishing the cellular epigenome, we examined whether loss of SMCHD1 function might affect muscle homeostasis through additional mechanisms. Here we show that acute depletion of SMCHD1 results in a DUX4-independent defect in myoblast proliferation. Genomic and transcriptomic experiments determined that SMCHD1 associates with enhancers of genes controlling cell cycle to activate their expression. Amongst these cell cycle regulatory genes, we identified LAP2 as a key target of SMCHD1 required for the expansion of myoblasts, where the ectopic expression of LAP2 rescues the proliferation defect of SMCHD1-depleted cells. Thus, the epigenetic regulator SMCHD1 can play the role of a transcriptional co-activator for maintaining the expression of genes required for muscle progenitor expansion. This DUX4-independent role for SMCHD1 in myoblasts suggests that the pathology of FSHD2 may be a consequence of defective muscle regeneration in addition to the muscle wasting caused by spurious DUX4 expression.

## Graphical Abstract

**Graphical Abstract:**
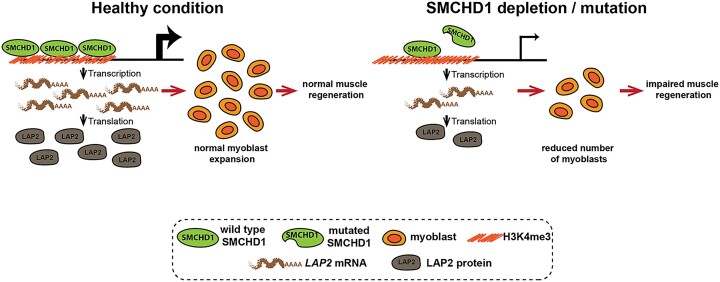

## Introduction

The *structural maintenance of chromosomes flexible hinge domain containing 1* (*SMCHD1*) gene encodes an epigenetic modifier first identified in a screen for genes involved in transgene array silencing (1). SMCHD1 is best known for its role in X chromosome inactivation (XCI) where it plays an essential role in merging the intermediate compartments to ensure proper gene silencing (2,3). SMCHD1 contributes to the establishment of heterochromatin by regulating DNA methylation, where it is required for Dnmt3b-mediated methylation of CpG islands on the inactive X chromosome (4), while also inhibiting the activity of Tet DNA demethylases (5). SMCHD1 can also actively repress gene transcription by limiting long-range chromatin interactions where it acts to deplete CTCF and cohesin from the topologically associated domain (TAD) boundaries at specific loci, including the Hox and protocadherin gene clusters (3,6–8).

Loss of function mutations in the *SMCHD1* gene are observed in a subgroup of patients with the muscle-wasting condition Facioscapulohumeral Muscular Dystrophy (FSHD), one of the most frequent neuromuscular diseases. However, the predominant cause of FSHD is a deletion of the subtelomeric D4Z4 tandem DNA array on chromosome 4. This contraction of the *D4Z4* locus (10 or less repeats) induces an epigenetic de-repression of the locus, which results in the ectopic expression of an RNA encoding double homeobox 4 (DUX4), a transcription factor whose presence in muscle fibers induces apoptosis (9–11). Based on this mechanism of action, it was later demonstrated that mutations reducing SMCHD1 function in muscle could lead to the de-repression of a slightly longer *D4Z4* tandem array (up to 16 repeats) to permit the expression of *DUX4* in patients with a variant of the disease called FSHD2 (12–14). Confirming the importance of *Dux4* expression as causal in FSHD, the onset of the muscle-wasting disease requires a patient to have a specific 4qA allele, which introduces a polyA signal downstream of the last copy of the *D4Z4* array to permit the expression of stable *DUX4* mRNA (15,16). Indeed, individuals that have a 4qB *D4Z4* allele will not develop FSHD despite possessing a *D4Z4* array contraction or an SMCHD1 mutation that opens chromatin at the Dux4 gene. These facts clearly establish that regardless of the mechanism of *D4Z4* locus de-repression, spurious accumulation of the DUX4 transcription factor protein is causal in FSHD. Nevertheless, patients who have mutations of *SMCHD1* in addition to a contraction of the *D4Z4* locus often display a more severe muscle-wasting phenotype (17,18). This raises the question of the potential for the general epigenetic silencer SMCHD1 to have additional roles in muscle cells that might exasperate the disease phenotype.

Here, we set out to investigate the function of SMCHD1 in human myoblasts that might affect the health of muscle independently of DUX4 expression. For this, we took advantage of the fact that the susceptibility of individuals to FSHD depends upon a specific polymorphism within the *D4Z4* locus since the 4qA chromosome variant creates a polyadenylation signal which is necessary to stabilize *DUX4* transcripts (16,19). We thus depleted SMCHD1 in cells derived from a healthy individual with a 4qB polymorphism at the *D4Z4* locus on both chromosomes that cannot give rise to stable *DUX4* transcripts (20). Using this approach, we uncovered a previously unappreciated DUX4-independent role for SMCHD1 as a co-activator of genes involved in cell cycle progression during myogenic progenitor cell expansion.

## Materials and methods

### Reagents and software

The names, sources, and catalogue numbers of the reagents used in the experiments are listed in Supplementary Table S1. All the software and databases used in the data analyses are listed in Supplementary Table S2.

### Cell culture

Immortalized non-FSHD myoblast cell line WS234 (donor 15Vbic) (20) was obtained from the Wellstone Muscular Dystrophy Cooperative Research Center for FSHD, University of Massachusetts Medical School, USA. This cell line was previously tested to possess a 4qB D4Z4 chromosome variant on both chromosomes and have no detectable *DUX4* mRNA expression (21). In proliferating condition, cells were cultured in LHCN medium [4:1 DMEM : Medium 199 supplemented with 15% BGS (v/v), 0.03 ug/ml Zinc Sulfate, 1.4 ug/ml vitamin B12, 0.055 ug/ml dexamethasone, 2.5 ng/ml HGF, 10 ng/ml FGF, 0.6× penicillin/streptomycin, 20 mM HEPES (pH 7.4)] on plates coated with 0.1% gelatin (w/v) at 37°C in 5% CO_2_ incubator. HEK293T cells were cultured in DMEM growth medium [DMEM supplemented with 10% BGS and 1× penicillin/streptomycin], at 37°C in 5% CO_2_ incubator.

### Plasmid generation

The plasmid vector pDONR223 containing the open reading frame of human *LAP2β* was purchased from the genome editing and molecular biology facility of the University of Ottawa. The open reading frame of *LAP2β* was amplified by PCR using the forward primer 5′-catggaggatccgccaccatgccggagttcctggaag-3′ and the reverse primer 5′-cgtatggtacctcattagttggattttctagggtca-3′. The resulting PCR product was cloned in the lentiviral vector pLenti-EF1a-Blank and the sequence of *LAP2β* was confirmed by Sanger sequencing.

### Preparation of lentivirus

HEK293T cells were transfected with the pMD2.G, psPAX2 and shRNA vector plasmids (Supplementary Table S3) using polyethyleneimine as previously described (22). Lentivirus-containing medium supernatant was centrifuged at 126 086 × g for 2.5 h at 4°C using Optima L-100 XP ultracentrifuge (Beckman Coulter), and the pellet was resuspended in LHCN medium. To transduce the cells, purified lentivirus and 8 μg/ml of polybrene was added to the culture medium. The cells were incubated for 16 hours at 37°C and then fresh LHCN medium was replaced.

### Growth curve, EdU proliferation essay

The cell proliferation rate was determined by EdU incorporation in newly synthesized DNA as well as cell counting. Three days after lentiviral transduction, an equivalent number of cells were seeded on each well of a gelatin-coated six-well plate. Fresh LHCN medium was replaced every 2 days. Five days after lentiviral transduction, 10 μM EdU was added to the culture medium for 90 min at 37°C. Cells were fixed to perform immunostaining according to the manufacturer's protocol of the EdU staining kit. For cell counting experiments, cells were trypsinized and resuspended LHCN medium. Trypan blue solution was added and the mixture was loaded to a hemocytometer for cell counting. The cell number from each well was calculated from the average of 6 independent hemocytometer counting.

### RNA-Seq and data analysis

Five days after *SMCHD1* or non-silencing lentiviral transduction, RNA was collected using a PureLink RNA mini kit according to the manufacturer's protocol. The depletion of rRNA was performed using the Ribo-Zero Magnetic Gold Kit, and the RNA library was prepared using KAPA stranded RNA-Seq kit according to the manufacturer's protocol. Sequencing was performed on an Illumina HiSeq4000 platform. RNA-Seq reads from fastq files were aligned to human hg38 genome using the STAR (23), counts were summarized using featureCounts function (24) in Rsubread and used as input for DESeq2 (25) to identify the differentially expressed genes. RNA-Seq datasets (26) downloaded from GEO were analyzed using the same settings as the SMCHD1 depletion RNA-Seq dataset. Volcano plots were made using EnhancedVolcano (https://github.com/kevinblighe/EnhancedVolcano), dot plots were made using ggplot2 (27) and heatmaps using pheatmap (https://github.com/raivokolde/pheatmap) in R. Gene ontology analysis was performed using DAVID (28), and values were used as inputs for dot plots.

### Gene set enrichment analysis

The down-regulated or up-regulated genes after SMCHD1 knockdown (adjusted *P* <= 0.05) were used as two separate gene set for Gene Set Enrichment Analysis (GSEA) (29). The analysis was performed using the GSEA software according to the user guide with the number of permutations set to 1000.

### Binding and expression target analysis (BETA)

The analysis was done following the user manual (30). Peaks within 150 kb of TSS and differentially expressed genes with adjusted *P* value ⇐ 0.05 were selected for the analysis, without using CTCF as a boundary to filter peaks around genes.

### ChIP-Seq and data analysis

The SMCHD1 ChIP-Seq was performed as previously described (31) with some modifications. Briefly, proliferating WS234 myoblasts were fixed in 1% (w/v) formaldehyde for 20 min at room temperature and quenched with 125 mM glycine for 10 min at room temperature. Cells were rinsed with PBS, collected using cell scrapers, centrifuged, and resuspended in swelling buffer [50 mM Tris base (pH 8.0), 300 mM sucrose, 10 mM NaCl, 0.5% NP-40 (v/v)]. Cells were incubated for 30 min in swelling buffer at 4°C, and sonicated using Bioruptor (Diagenode) for 2 cycles (HI power, cycles of 30 s on/1 min off). Nuclei were spun down at 1000 xg for 5 min at 4°C, resuspended in sonication buffer [50 mM HEPES (pH 7.9), 140 mM NaCl, 1 mM EDTA, 1% Triton X-100 (v/v), 0.1% Na-deoxycholate (w/v),1% SDS (w/v)], and sonicated for 13 cycles using the same settings as the previous step. Next, 7.5 ug of SMCHD1 antibody or normal rabbit IgG was added to 250 ug of precleared chromatin and incubated for 3 h at room temperature. The chromatin was then washed and purified as described previously (31). Library was prepared using Kapa Hyperprep kit and paired-end sequencing was performed on an Illumina HiSeq4000 platform. The reads were aligned to the human hg38 genome using bowtie2 (32) and peaks were called by MACS2 (33) using a *q* value cutoff = 0.05. Meta profile plots of ChIP-Seq signal around SMCHD1 peaks were generated using EnrichedHeatmap and ggplot2 in R.

### CUT&Tag and data analysis

Analysis of histone modification enrichment across the genome was performed using CUT&Tag as previously described (34). Antibodies against either H3K4me3, H3K4me1, H3K9me3, H3K27ac or H3K27me3 (or control of rabbit IgG) were incubated with digitonin permeabilized WS234 myoblasts. After washing away the unbound antibodies, permeabilized cells were then incubated with the Tn5-PA protein. After a subsequent wash, cells were incubated with MgCl_2_ to induce tagmentation of the chromatin at sites enriched for the specific histone marks of interest. Tagmented DNA was amplified by PCR to generate a library for sequencing on an Illumina NovaSeq 6000 with 100 bp paired-end reads to a depth of 10M reads per library. Reads were aligned to the human hg38 genome using Bowtie2 (32) and peaks were called by MACS2 (33) using a *q* value cutoff = 0.05.

### Motif identification using HOMER (Hypergeometric Optimization of Motif EnRichment)

The bed files generated by MACS2 were converted to fasta files using ‘getfasta’ function in BEDTools (35). The converted fasta files were used as input files for HOMER analysis (36) using the ‘findMotifs.pl’ function.

### Statistical analyses

For RNA-Seq, differentially expressed genes were defined as genes with a fold change >0.5 and an adjusted *P*-value <= 0.05, unless otherwise specified. For the identification of the ChIP-Seq peak identification using MACS2 (33), the cutoff value q value cutoff = 0.05. For overlapping ChIP-Seq peaks between two datasets, *P* values and *Z* scores were calculated by permutation test using regioneR (37) with number of permutations = 1000. Two-way ANOVA (for cell counting experiments) and Student's two-tailed *t*-test (for other experiments) were performed using the statistical graphing software GraphPad Prism v6.0, and *P< 0.05, **P< 0.01, ***P< 0.001, ****P< 0.0001, ns = not significant.

## Results

### SMCHD1 is required for the efficient proliferation of human myoblasts

**Figure 1.**
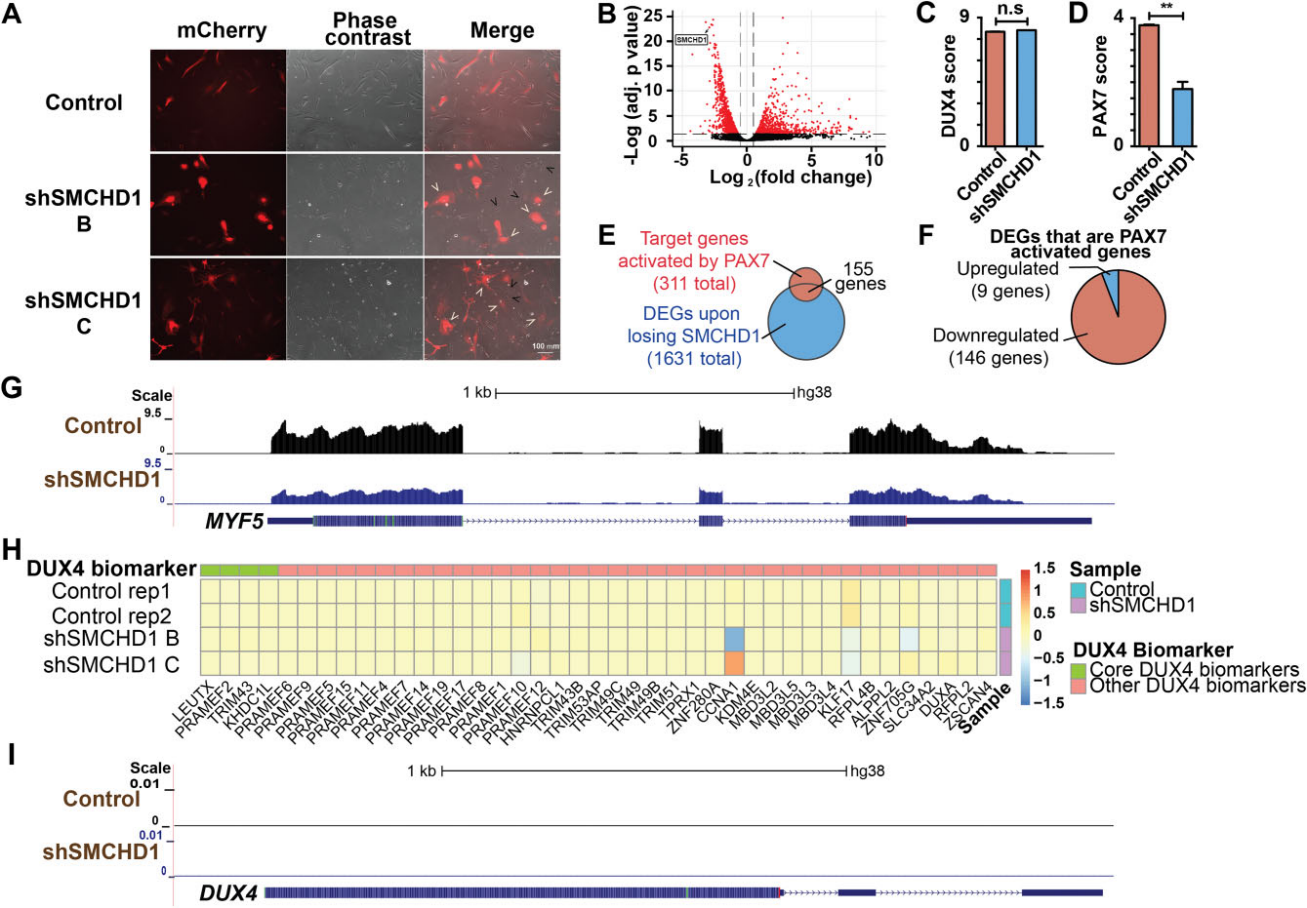
Loss of SMCHD1 leads to FSHD-like repression of PAX7 target genes in myoblasts without DUX4 target genes activation. (**A**) Nine days after lentiviral transduction, cells were observed under the fluorescence microscope. (White arrows) Transduced mCherry^+^*SMCHD1* shRNA expressing cells. (Black arrows) Cells that do not express shRNA expression. (**B**) Volcano plot showing the DEGs after SMCHD1 knockdown. Genes with adjusted *p*-value <= 0.05 and log_2_ fold change > 0.5 were labeled red. (**C**) DUX4 score of samples transduced with non-silencing scrambled shRNA (Control) or *SMCHD1* targeting shRNA (shSMCHD1) based on DUX4 targets identified by Choi *et al.* (39). (**D**) PAX7 scores of samples transduced with non-silencing scrambled shRNA (Control) or *SMCHD1* targeting shRNA (shSMCHD1). (**E**) Venn diagram of the target genes activated by PAX7 and differentially expressed upon SMCHD1 depletion. (**F**) Number of PAX7-activated genes activated by PAX7 that were down-regulated (red) and up-regulated (blue) upon SMCHD1 depletion. (**G**) UCSC genome browser tracts showing RNA-Seq reads of samples transduced with non-silencing scrambled shRNA (Control) or *SMCHD1* targeting shRNA (shSMCHD1) near the *MYF5* gene. (**H**) Heatmap showing the expression of DUX4 target genes identified by Yao et. al. (41) upon SMCHD1 depletion. (**I**) UCSC genome browser tracts showing RNA-Seq reads of samples transduced with non-silencing scrambled shRNA (Control) or *SMCHD1* targeting shRNA (shSMCHD1) near the *DUX4* gene.

To determine the roles of SMCHD1 that are independent of DUX4 in adult myogenesis, we performed an acute depletion of SMCHD1 in immortalized human WS234 myoblasts (20). WS234 cells were chosen as they possess the non-pathogenic 4qB *D4Z4* allele on both copies of chromosome 4, preventing the expression of a stable *DUX4* mRNA transcript (21). We opted to use a shRNA-mediated approach for depletion to prevent the selection of clones that had adapted to the complete loss of SMCHD1. Using three different shRNAs targeting distinct regions of the *SMCHD1* transcript, we observed a reduction in mRNA and protein accumulation that varied between 50 and 90% when examined 5 days after lentivirus infection (Supplementary Figure S1A and S1B). These reduced levels of SMCHD1 would be consistent with the haploinsufficiency in FSHD2 patients possessing a heterozygous mutation of the gene. Phenotypically, the reduction in SMCHD1 levels in cells caused the muscle progenitor cells to adopt a less elongated morphology compared to the controls (Figure 1A). This change in cell appearance suggests that SMCHD1 may be important for myoblast function.

### Loss of SMCHD1 leads to aberrant expression of PAX7 target genes in myoblasts

To understand how the loss of SMCHD1 impairs myoblast function, we performed RNA-sequencing analysis on myoblasts depleted of SMCHD1 using two distinct shRNAs. Analysis of gene expression changes in myoblasts depleted of SMCHD1 identified 1631 differentially expressed genes (932 down-regulated and 699 up-regulated) (Figure 1B and Supplementary File S1). Though we were using myoblasts that possess only the 4qB *D4Z4* allele, we wanted to confirm the absence of DUX4 in our myoblasts. Indeed, no transcripts of *DUX4* were detected in our deep-sequencing of RNA from WS234 cells under any conditions tested. Due to the difficulty of detecting *DUX4* transcripts in muscle cells, measurement of DUX4-target gene expression is often used as a surrogate for detecting the presence of DUX4 (38). Measuring the previously described ‘DUX4 score’ (38), we observed that SMCHD1 knockdown did not significantly change the expression of previously established DUX4 biomarkers (39–41) (Figure 1C and Supplementary Figure S1C). Reasoning that the loss of SMCHD1 may only change the expression of the most robust DUX4-FSHD biomarkers, we re-assessed a DUX4 score using only the top biomarkers identified by Yao et al. (41). Again we found that the depletion of SMCHD1 did not significantly change the expression of the four core sets of DUX4-FSHD biomarkers (Figure 1H). Thus, the proliferation defects observed in the absence of SMCHD1 are not the result of induced DUX4 expression.

A hallmark of myoblasts from FSHD patients is the repression of Pax7 target genes (42). As such, we next examined whether the expression of known Pax7 target genes was altered upon depletion of SMCHD1. For this purpose, we calculated a PAX7 score (38) based on changes in transcript abundance upon SMCHD1 depletion (Figure 1D). We observed that ∼50% of the genes normally activated by Pax7 show reduced expression in myoblasts depleted of SMCHD1 (Figure 1E–G), while ∼11% of genes repressed by PAX7 expression show increased expression upon SMCHD1 depletion (Supplementary Figure S1D, E). Therefore, our data showed that SMCHD1 depletion reproduces some of the altered gene expression as observed in myoblasts-derived from FSHD patients despite the fact that DUX4 itself is not expressed (Figure 1I).

**Figure 2.**
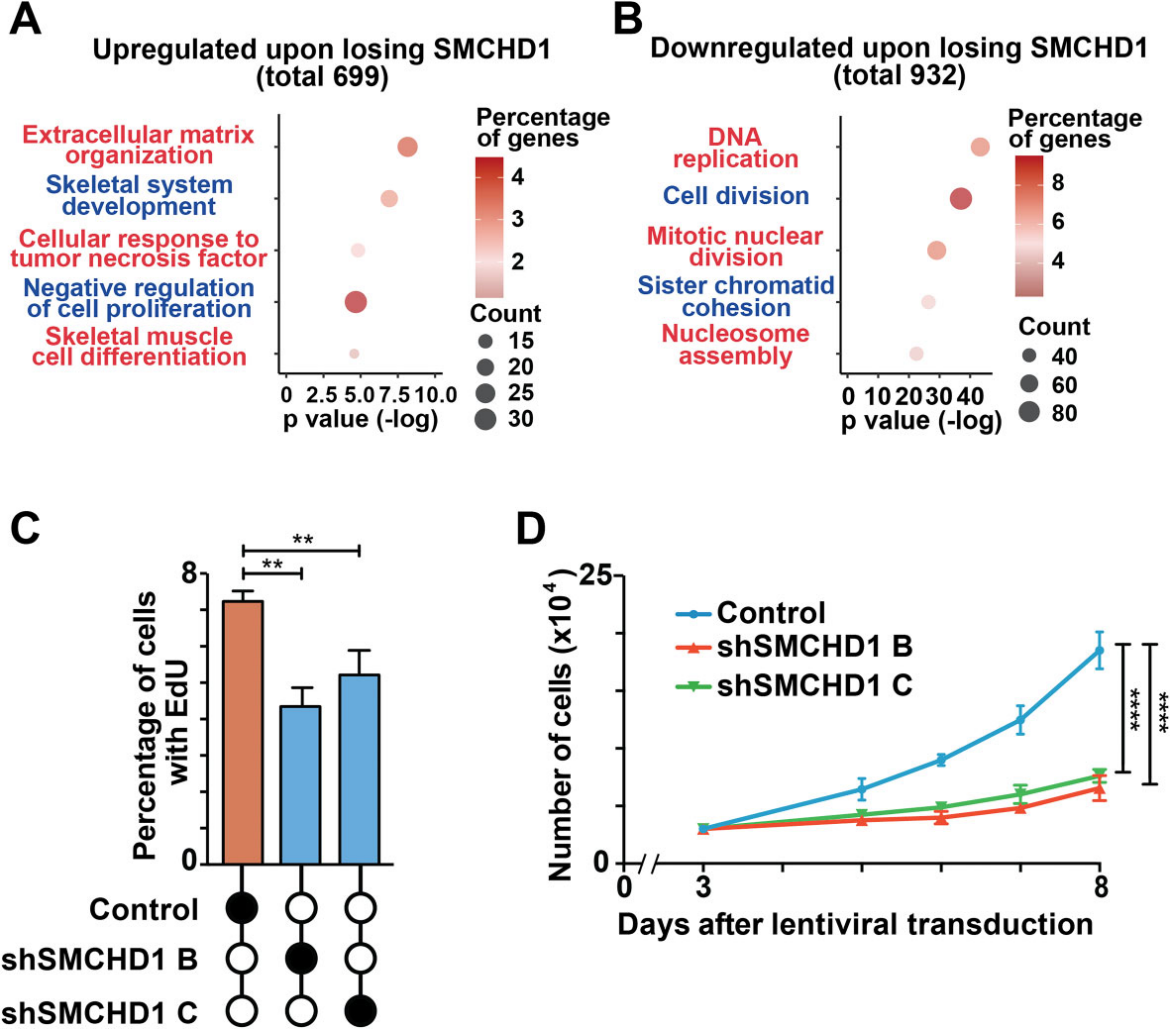
Loss of SMCHD1 in myoblasts leads to a decrease in proliferation. (**A**) GO terms of up-regulated genes after SMCHD1 depletion. (**B**) GO terms of down-regulated genes after SMCHD1 depletion. (**C**) Five days after lentiviral transduction, an EdU cell proliferation assay was performed, and the percentage of EdU^+^ cells was quantified. Error bars represent standard deviation from three independent experiments. (**D**) An equal number of myoblasts was seeded 3 days after lentiviral infection, and cell number was counted from day 5 to day 8 after lentiviral transduction. Error bars represent standard deviation from 3 independent experiments.

To better understand how the loss of SMCHD1 was affecting myoblast function, we performed gene ontology (GO) enrichment analysis. Consistent with results observed in FSHD patients with *SMCHD1* mutations (43), loss of the epigenetic modifier in myoblasts results in the upregulation of genes associated with the extracellular matrix (Figure 2A and Supplementary Figure S2A). Though SMCHD1 is known as an epigenetic silencer, we were intrigued by the large number of genes involved in cell cycle (DNA replication, nucleosome assembly and G1/S transition) that were down-regulated upon loss of SMCHD1 (Figure 2B). Using RT-qPCR, we confirmed that SMCHD1-depletion leads to the downregulation of the genes coding for cyclins and cyclin-dependent kinases (Supplementary Figure S2B), centromeres (Supplementary Figure S2C) and core histone proteins (Supplementary Figure S2D). These findings suggest that loss of SMCHD1 reduces the expression of genes associated with cell cycle progression and may affect the proliferation of myoblasts. To explore this possibility, we examined the expansion of SMCHD1-depleted myoblasts (Figure 2C-D). Measuring the rate of EdU incorporation into newly synthesized DNA, we observed that loss of SMCHD1 resulted in fewer myoblasts passing through S-phase of the cell cycle (Figure 2C). This decreased passage through cell cycle resulted in a reduced expansion of the progenitor population as determined by direct counting of cell numbers over time (Figure 2D). Taken together this data indicates that the loss of SMCHD1 in human myoblasts leads to a decreased ability of the progenitor population to expand their numbers.

**Figure 3.**
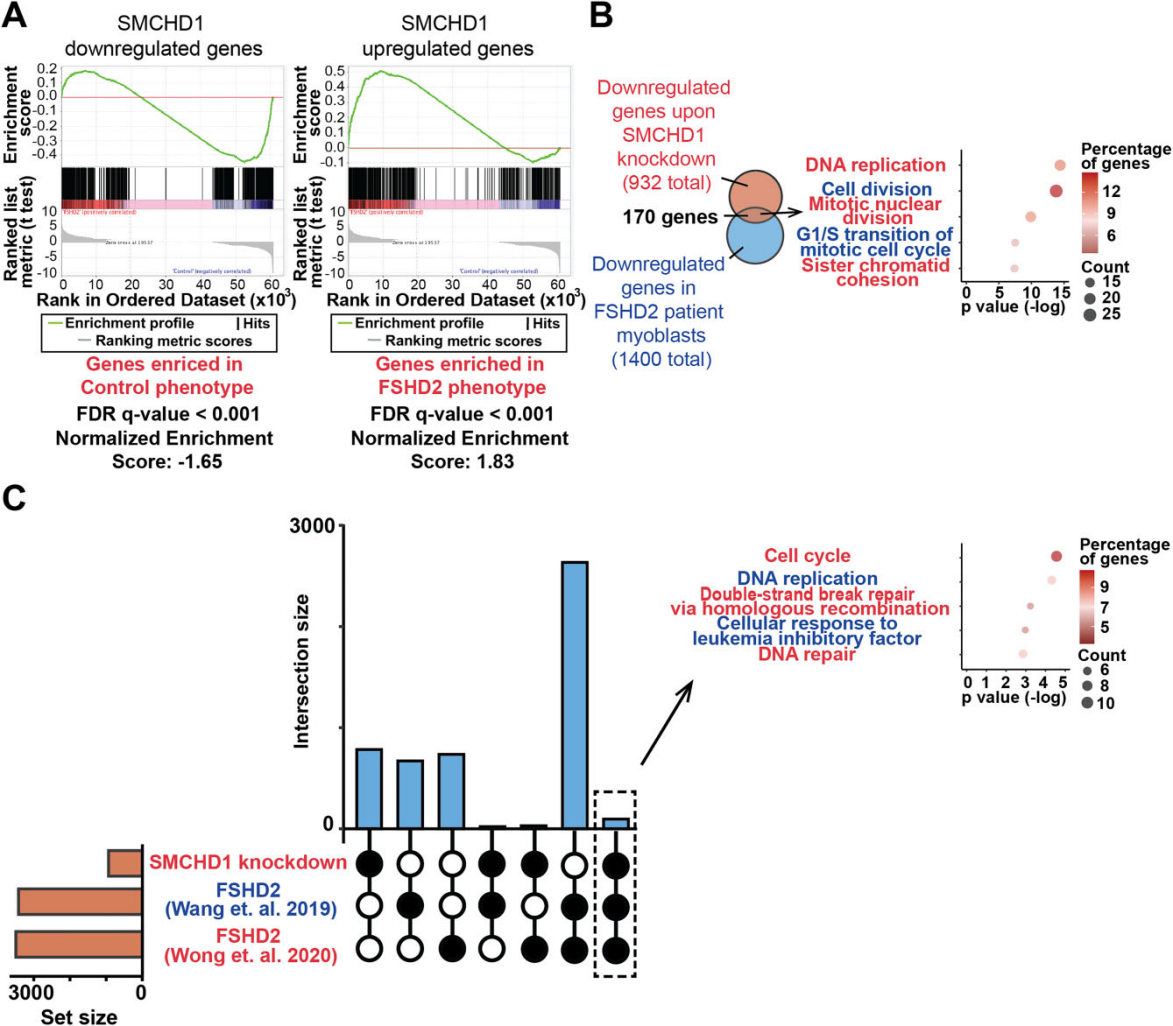
Comparison of changes in gene expression profiles upon SMCHD1 depletion and under FSHD2 condition. (**A**) Gene set enrichment analysis (GSEA) of DEGs in FSHD2 myoblasts compared to down-regulated (left panel) or up-regulated (right panel) genes upon SMCHD1 depletion. (**B**) (Left panel) Venn diagram of commonly down-regulated genes and (Right panel) dot plot of the top GO terms of the genes commonly down-regulated in SMCHD1-depleted myoblasts and in FSHD2 myoblasts. (**C**) (Left panel) Upset plot showing the commonly down-regulated genes upon SMCHD1 depletion and in FSHD2 biopsies from Wang *et al.* (45) and Wong *et al.* (43) studies. (Right panel) GO terms of commonly down-regulated genes upon SMCHD1 depletion and in FSHD2 biopsies.

Having observed that loss of SMCHD1 results in FSHD-like changes in PAX7 target gene expression, we next examined whether the down-regulation of cell cycle genes observed upon depletion of SMCHD1 in myoblasts might be an unappreciated defect in myoblasts from FSHD patients with SMCHD1 mutations. Published datasets from FSHD patients with D4Z4 contractions (42,44) or SMCHD1 mutations (26) were analyzed in combination with RNA-Seq data from our SMCHD1-depleted myoblast using Gene Set Enrichment Analysis (GSEA). Since these studies looked at differentiation of primary myoblasts, we limited our comparative analysis to the 0h time point (ie proliferation) of these published datasets (26,42,44). Interestingly, we observed that genes that change their expression (both up- or down-regulated) in myoblasts upon loss of SMCHD1 overlapped significantly with genes that change their expression in FSHD myoblasts compared to healthy controls (Figure 3A and Supplementary Figure S3A). This strong correlation between FSHD patient myoblasts and myoblasts depleted of SMCHD1 suggested these cells may share similar defects, and led us to examine whether the expression of cell cycle genes was also dysregulated in FSHD patients. GO analysis showed that FSHD2 myoblasts from patients have altered expression of genes involved in cell division, and DNA replication (Supplementary Figure S3B), as well as negative regulation of cell proliferation (Supplementary Figure S3C). Unexpectedly, we observed that the changes in gene expression related to cell cycle and cell proliferation were specific to FSHD2 (Figure 3B), but not in FSHD1 myoblasts (Supplementary Figure S3D-F). The defect in cell cycle gene expression could also be observed (Figure 3C) in biopsies taken from FSHD2 patients (43,45). Thus, down-regulation of genes involved in cell cycle progression is a characteristic shared between myoblasts depleted of SMCHD1 (myoblasts with shRNA-mediated depletion of SMCDH1 or primary FSHD2 patient myoblasts), but not myoblasts from FSHD patients that retain SMCHD1 (FSHD1). The similar changes in cell cycle gene expression between SMCHD1-depleted myoblasts and in FSHD2 patient samples was intriguing given that previous studies have demonstrated that the doubling time of healthy and FSHD1 myoblasts were similar (46,47).

### SMCHD1 binds at transcriptional regulatory regions of cell cycle genes in human myoblasts

SMCHD1 is known as a transcriptional silencer, yet the loss of SMDHD1 results in the down-regulation of cell cycle genes. This led us to examine whether genes down-regulated by the loss of SMCHD1 might be directly targeted by the epigenetic regulatory protein. Using chromatin immunoprecipitation sequencing (ChIP-Seq), we probed the genome-wide binding of SMCHD1 in WS234 cells. We identified 15 560 sites (*q* value <= 0.05) of binding of SMCHD1 in proliferating myoblasts (Supplementary File S2). Knowing that SMCHD1 is involved in X chromosome inactivation (2), we first examined the distribution of SMCHD1 across the different chromosomes. As expected, we found that SMCHD1 binding was enriched on the X chromosome, with ∼4 fold more SMCHD1 binding sites relative to individual autosomes (15.1 sites per Mb on X chromosome versus 3.6 sites per Mb on autosomes) (Supplementary Figure S4A). Similarly, we observed a strong enrichment of SMCHD1 (Supplementary Figure S4B-S4D) at sites previously known to be targeted by SMCHD1, including telomeres (48) as well as the 5S rRNA and tRNA clusters on chromosome 1 (49). This established that SMCHD1 in human myoblasts localizes to many of the regions previously described for other cell types and provides confidence in the specificity of our ChIP-Seq.

**Figure 4.**
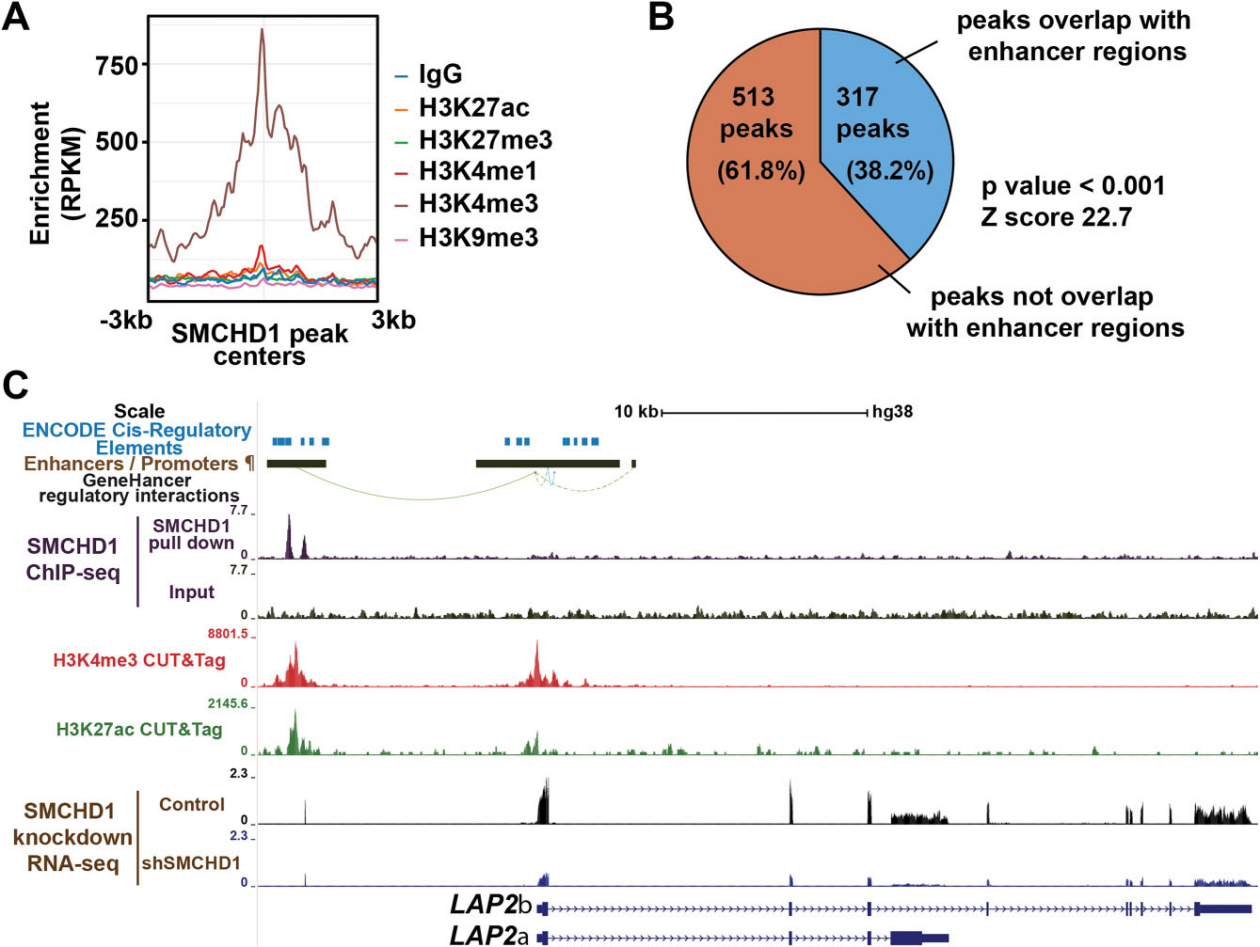
Binding regions of SMCHD1 in the human myoblast genome. (**A**) The enrichment of H3K9me3, H3K4me1, H3K4me3, H3K27me3 and H3K27ac around SMCHD1 peak centers at SMCHD1 co-activated genes. (**B**) Percentage of direct targets-associated peaks that were located at promoters and enhancers. *Z* score and *P*-value were calculated by permutation test using regioneR. (**C**) UCSC genome browser tracts showing ENCODE *cis-*regulatory elements, GeneHancer annotated enhancer/promoter regions (¶) and their regulatory interactions, as well as ChIP-Seq (SMCHD1), CUT&Tag (H3K4me3 and H3K27ac) and RNA-Seq reads (control: proliferating myoblasts expressing non-silencing scrambled shRNA, shSMCHD1: proliferating myoblasts expressing shRNA targeting *SMCHD1*) near the *LAP2* gene.

We next wanted to associate SMCHD1 binding at specific genes to changes in gene expression in order to identify direct target genes. For this purpose, we performed Binding and Expression Target Analysis (BETA) which directly integrates data from ChIP-Seq and RNA-Seq (30). Using this approach, we identified 358 genes bound by SMCHD1 that changed their expression upon SMCHD1 depletion (Supplementary File S2). Though SMCHD1 has known silencing activity, we observed that many target genes are repressed upon loss of SMCHD1 (181 down-regulated and 177 up-regulated). We then asked whether SMCHD1’s binding motifs are different in those sites associated with down-regulated versus up-regulated targets. We found that the majority of the enriched motifs were similar between the two conditions (Supplementary Figure S5) with some exceptions. For example, the ZBTB3 motif of CCACTVYAC was specifically enriched at the peaks associated with SMCHD1 co-activated genes (Supplementary Figure S5A), while the ZNF416 motif of CCCAGCT was specifically enriched at peaks associated with SMCHD1 co-repressed genes (Supplementary Figure S5B). To identify epigenetic characteristics associated with SMCHD1 binding sites, we performed CUT&Tag analysis for various histone marks (H3K27ac, H3K9me3, H3K4me3, H3K4me1 and H3K27me3) in proliferating WS234 myoblasts. Globally we observed that SMCHD1 preferentially bound at heterochromatic regions enriched for H3K9me3 marks. This finding was expected and is consistent with the binding of SMCHD1 to the telomers and the inactive X chromosome, and work showing that the binding of SMCHD1 at the *D4Z4* locus is H3K9me3 dependent (50). However, when analysis was restricted to SMCHD1 peaks associated with genes that show reduced expression upon SMCHD1-depletion, we observed an enrichment for the transcriptionally permissive H3K4me3 mark but no enrichment of the repressive marks H3K9me3 or H3K27me3 (Figure 4A). This finding led us to examine whether SMCHD1 might be associated with transcriptional regulatory regions. Using the human GeneHancer database (51), our analysis determined that 38% of SMCHD1 direct target genes (Z score = 22.7) showed SMCHD1 binding at previously identified promoters or enhancers (Figure 4B). Consistent with previous findings (52) we observed the co-binding of CTCF at many of these SMCHD1-bound enhancers (Supplementary Figure S5C). This indicates a role for SMCHD1 in helping establish active enhancers at genes involved in cell cycle regulation.

### SMCHD1 regulates the proliferation of myoblasts via its direct target gene *LAP2*

**Figure 5.**
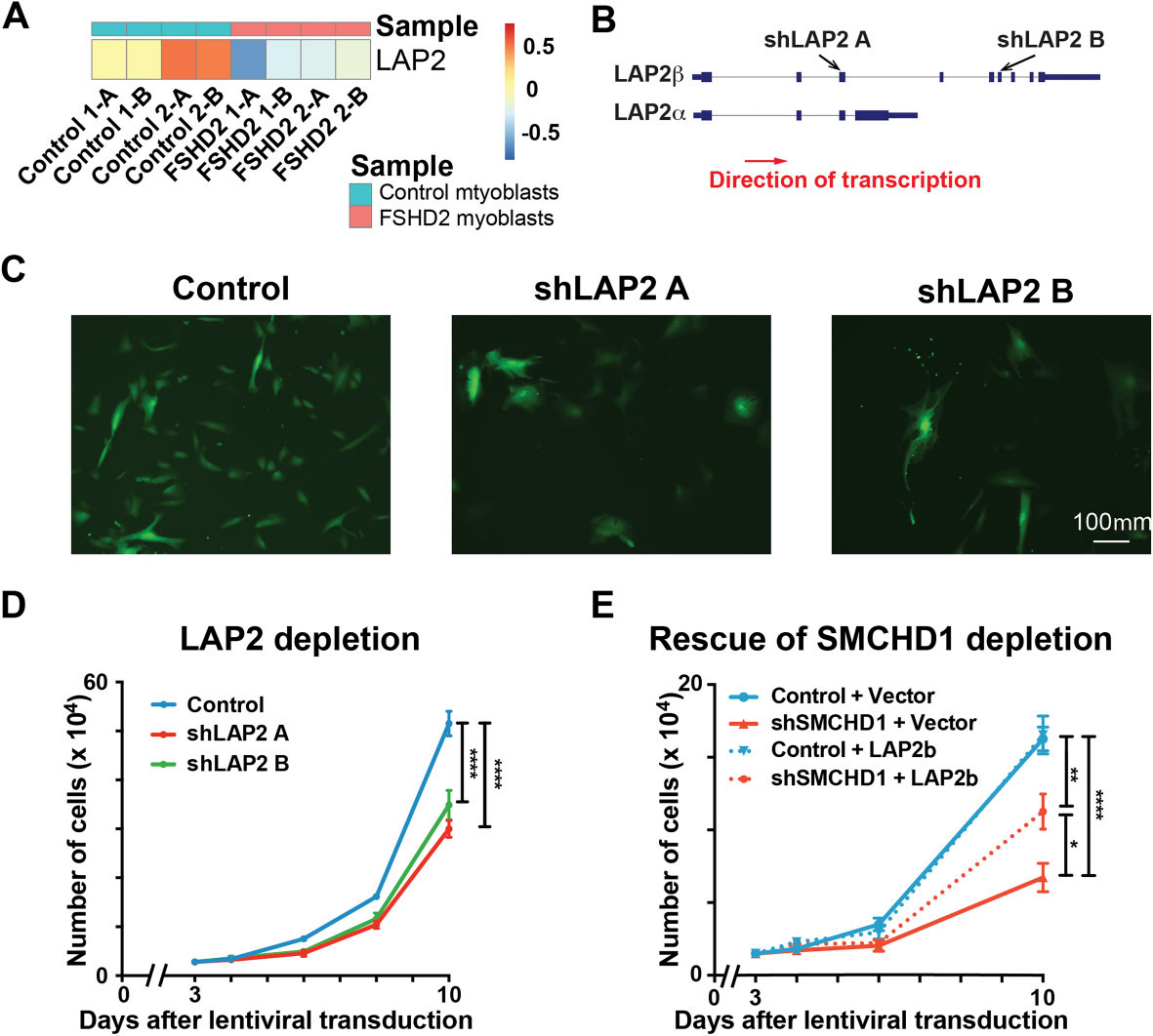
LAP2 depletion slows myoblast proliferation. (**A**) Heatmap showing the RNA expression of *LAP2* in FSHD2 and control non-FSHD myoblasts (GEO accession number GSE143493). (**B**) Different *LAP2* isoforms and target regions of different *LAP2* shRNAs. (**C**) Nine days after *LAP2* shRNA transduction, cells were observed under a fluorescence microscope for GFP^+^ shRNA-expressing cells. (**D**) Three days after *LAP2* depletion, an equal number of myoblasts were seeded on 6 well plates. Cell number was counted on day 4, 6, 8 and 10 after lentiviral transduction. Error bars represent standard deviation from 3 independent experiments. (**E**) Lentivirus was added to the culture medium of proliferating myoblasts as indicated (control: non-silencing scrambled shRNA, shSMCHD1: shRNA targeting *SMCHD1*, vector: plenti-GIII vector alone, LAP2β: plenti-GIII vector expressing LAP2β transcript). Three days after lentiviral transduction, equal number of myoblasts were seeded on six-well plates. Cell number was counted on days 4, 6 and 10 after lentiviral transduction. Error bars represent standard deviation from three independent experiments.

Having identified a list of genes involved in cell cycle regulation as direct targets of SMCHD1, we next sought to identify genes that contribute to the cell proliferation defect in SMCHD1-depleted cells. Amongst these cell cycle regulators directly regulated by SMCHD1, we focused on Lamina-associated polypeptide 2 (LAP2, also known as Thymopoietin or TMPO) (Figure 4C), since our analysis of published RNA-Seq data (26) showed the gene was down-regulated in proliferating primary myoblasts isolated from FSHD2 patients (Figure 5A) while being unaltered in myoblasts from FSHD1 patients (Supplementary Figure S6A). Among the different isoforms of the *LAP2* gene (Figure 5B), LAP2α is a lamin A/C binding protein that has been shown to be essential for the proliferation of glioblastoma cells (53), while LAP2β is essential for nuclear envelope reassembly after mitosis in *xenopus* (54). We therefore tested whether the loss of LAP2 would also lead to a proliferation defect in human myoblasts. We were able to obtain a strong depletion of LAP2 expression in myoblasts using two different shRNA targeting LAP2 (Figure 5B, and Supplementary Figure S6B). Examining cell morphology at 9 days after shRNA treatment, we observed that myoblasts depleted of LAP2 became more flattened and spread out compared to control cells (expressing non-silencing shRNA) which continued to maintain a normal myoblast morphology (Figure 5C). Counting the cell numbers as the population proliferated over 10 days, myoblasts depleted in LAP2 showed a reduced rate of proliferation (Figure 5D). This indicates that the effect of losing SMCHD1 on the proliferation and morphology of myoblasts can be recapitulated by the loss of LAP2. Examination of exon inclusion from RNA-seq data indicated that LAP2β is the predominant isoform of the gene expressed in proliferating human myoblasts (Figure 4C). To determine whether LAP2 could rescue the SMCHD1 depletion-mediated proliferation defect, we overexpressed LAP2β in myoblasts depleted of SMCHD1. Excitingly, ectopic expression of LAP2β was sufficient to significantly improve the proliferation of SMCHD1-depleted myoblasts (Figure 5E). In summary, our data suggest that SMCHD1 helps ensure the efficient proliferation of human myoblasts by activating the expression of its target genes involved in cell cycle progression, including LAP2.

## Discussion

The detrimental effects of reduced SMCHD1 levels in mature muscle myofibers have been shown to be at the heart of the FSHD disease pathology in FSHD2 patients. However, the importance of the ubiquitously expressed SMCHD1 protein in maintaining and expanding the myoblast population has yet to be fully established. Here, we show that SMCHD1 is required for the efficient expansion of muscle progenitor cells. We found that myoblasts depleted of SMCHD1 show a reduced rate of proliferation that correlates with reduced expression of cell cycle-related genes at the transcription level. While these changes in gene expression occur in the absence of DUX4 expression, it is notable that the same dysregulation of cell cycle gene expression can be observed in muscle progenitor cells obtained from FSHD2 patients with SMCHD1 mutations. This is consistent with FSHD being a proposed satellite cell-opathy (55,56) and suggests that impaired expansion of myoblasts during regeneration may contribute to the disease pathology in FSHD2 patients.

The application of a knockdown approach to diminish SMCHD1 levels has allowed us to delineate a role for the chromatin-modifying enzyme in regulating the proliferation of human muscle progenitor cells. The reduced expansion of myoblasts upon reduction of SMCHD1 levels is associated with the down-regulation of genes involved in DNA replication and cell cycle progression. Consistent with the role of SMCHD1 in promoting cell proliferation, it has been shown that SMCHD1 is required for the expression of cell cycle genes in the early mouse embryo and that depletion of maternal SMCHD1 in the oocyte reduces the number of cells present in the blastocyst (57). Similarly, deletion of SMCHD1 in the hematopoietic system resulted in hematopoietic stem cells (HSCs) that were inefficient at competing with wild-type HSCs for repopulating the bone marrow niche (58). This suggests that SMCHD1 plays a role in the expansion of various stem cell populations.

**Figure 6.**
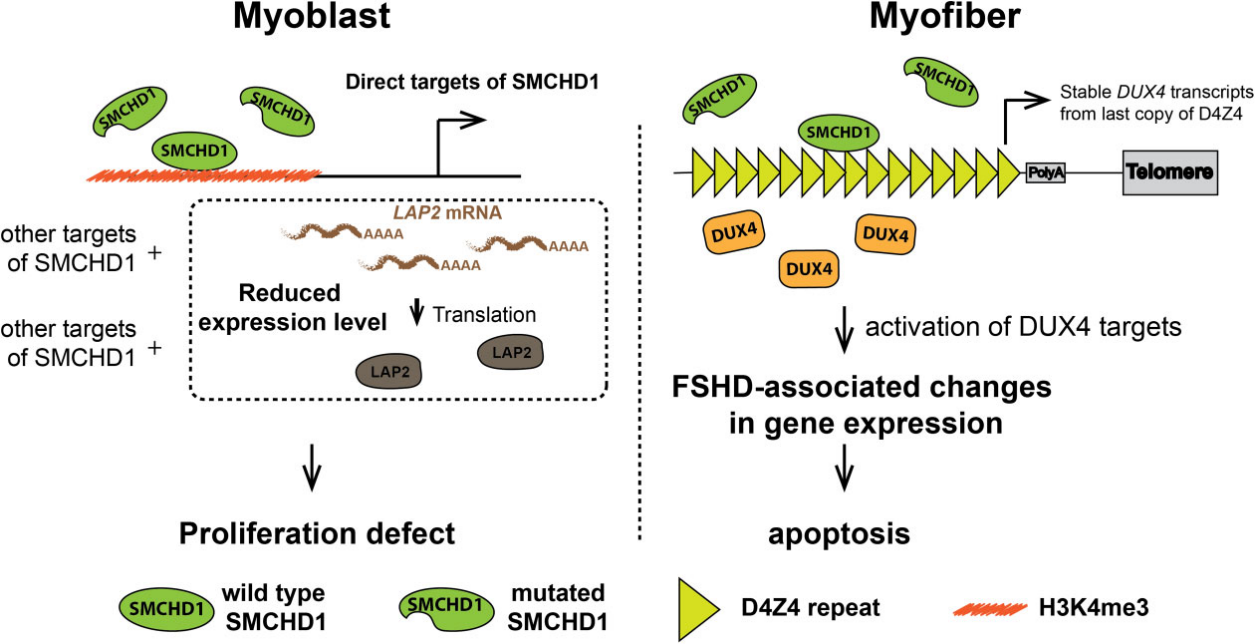
Working model of how the loss of SMCHD1 function results in FSHD2-associated defects. (Left panel) Loss of SMCHD1 binding on the promoter and enhancer regions of its target genes in myoblasts may result in additional problems to myoblasts growth, on top of those caused by spurious DUX4 expression. (Right panel) Heterozygous SMCHD1 mutations in FSHD2 patients lead to a reduced SMCHD1 binding on the D4Z4 locus and the expression of DUX4 in muscle, which results in the degeneration of myofiber.

We identified *LAP2* as a key SMCHD1-target gene that ensures efficient proliferation of the myoblasts. Indeed, SMCHD1 binds to an enhancer for *LAP2*, and is required for efficient expression of this protein that links the nuclear envelope to chromatin. The LAP proteins have been shown to play an important role in cell proliferation through the regulation of Rb/E2F1 activity as well as through the re-organization of nuclear envelope-associated proteins after cell division (59). Importantly, we demonstrated that exogenous expression of the LAP2β isoform was able to partially rescue the proliferation defect observed in conditions of diminished SMCHD1 levels. Our findings are particularly relevant for patients with FSHD2 since the diminished expression of *LAP2* was also observed in individuals carrying SMCHD1 mutations. Although it is generally accepted that FSHD2 patients do not present unique clinical phenotypes (60), we observed that muscle progenitor cells from FSHD2 patients show reduced expression of many of the cell cycle-related genes that were identified upon SMCHD1 depletion. As such, patients with mutations in SMCHD1 might expect to show diminished regeneration efficiency in addition to the loss of muscle due to spurious DUX4 expression. Though patients with FSHD1 have been shown to have defective regeneration due to impaired muscle differentiation (56), our identification of this DUX4-independent role for SMCHD1 in muscle regeneration (Figure 6), provides an additional explanation for previous findings that SMCHD1 mutations can act as a disease modifier that increases the disease severity FSHD1 patients with D4Z4 locus contractions that cause muscle wasting (17). In these patients, not only would the muscle fibers undergo wasting but the muscle would also have a reduced ability to regenerate due to poor expansion of the muscle progenitors. Thus, we have identified a key role for SMCHD1 in maintaining the expression of genes involved in the proliferation of muscle progenitor cells.

A surprising finding from our study was the determination that SMCHD1 plays the role of a transcriptional activator in certain contexts. SMCHD1 is classically known for its role in silencing large chromatin domains (1). During the onset of X-chromosome inactivation, recruitment of SMCHD1 to the intermediate compartments along the chromosome allows their fusion to form repressive megadomains that ensure gene silencing (3). As a non-classical SMC protein, it is thought that SMCHD1 creates these repressive megadomains by entrapping DNA in dense loops of chromatin (61). However, we and others have observed that depletion of SMCHD1 results in a large number of genes being down-regulated, a result consistent with a transcriptional co-activator function (this study; 57–58). As expected, genome-wide analysis of SMCHD1 binding showed an accumulation of the epigenetic modifier at sites-specific repressive domains in myoblasts. Unexpectedly, we also observed that SMCHD1 binds at transcriptional enhancers and/or promoters for many of the genes that are down-regulated upon removal of the epigenetic modifier. This is consistent with SMCHD1 acting as a transcriptional co-activator in certain genomic contexts. Of note, we found that ZBTB3 motifs are specifically enriched at SMCHD1 binding sites associated with co-activated genes. This is interesting since ZBTB3 is known as a structural regulator of enhancer-promoter interactions (62) where it co-activates genes to modulate the self-renewal of embryonic stem cells (63) and the proliferation of cancer cells (64). The enrichment of ZBTB3 motifs at these co-activated genes suggests that this site-specific DNA-binding factor could be responsible for recruiting SMCHD1 to selected genes, where SMCHD1 then acts as the ‘motor’ to mediate the loop extrusion that brings the enhancers in contact with the promoters to facilitate transcriptional elongation. Future studies will be required to examine the relationship between SMCHD1 and ZBTB3 in co-activating cell cycle genes in proliferating myoblasts. Importantly, SMCHD1 is not the first example in which an epigenetic regulator has been found to have a dual role in regulating gene expression. While the polycomb repressive complexes PRC1 and PRC2 play an important role in maintaining transcriptional repression at the *D4Z4* locus (65–67), it has been shown that the binding of PRC1 (68–70) and PRC2 (71,72) at enhancers can also promote the activation of developmental gene expression by establishing long-range chromatin interactions. Thus, it appears that multiple different transcriptional repressors that mediate long-range interactions can be co-opted to facilitate the activation of gene expression in certain contexts.

In summary, we have identified a role for SMCHD1 in modulating the expansion of muscle progenitor cells through the activation of genes that control cell cycle progression. These findings suggest that impaired muscle regeneration may contribute to the disease pathology in FSHD2 patients with mutations that induce SMCHD1 haploinsufficiency.

## Supplementary Material

gkae600_Supplemental_Files

## Data Availability

The RNA-Seq and ChIP-Seq data sets generated during this study are available in Gene Expression Omnibus as GSE190830, while CUT&Tag data sets are available as GSE267354.

## Notes

Present address: Matthew Man-Kin Wong, Department of Genetics, Stanford University, Palo Alto, CA 94304, USA.
