# Supplementary material for: SMCHD1 activates the expression of genes required for the expansion of human myoblasts
Source: Nucleic Acids Res. 2024 Jul 12;52(16):9450–62. doi: 10.1093/nar/gkae600 (PMC11381350; doi:10.1093/nar/gkae600)
Supplement: gkae600_Supplemental_Files [file gkae600_supplemental_files.zip › Wong Supplementary Data.pdf]

**Supplemental Data**

# SMCHD1 activates the expression of genes required for the expansion of human myoblasts

Matthew Man-Kin Wong[1,2], Sarah Hachmer[3], Ed Gardner[1,2], Valeria Runfola[5], Eric Arezza[1], Lynn A. Megeney[1,2], Charles P. Emerson Jr[4], Davide Gabellini[5], and F. Jeffrey Dilworth[1,2,3*]

[1]Sprott Center for Stem Cell Research, Regenerative Medicine Program, Ottawa Hospital Research Institute; Ottawa, ON, K1H 8L6, Canada

[2]Department of Cellular and Molecular Medicine, University of Ottawa; Ottawa, ON, K1H 8L6, Canada.

[3]Department of Cell and Regenerative Biology, University of Wisconsin; Madison, WI, 53705, USA

[4]Wellstone Muscular Dystrophy Program, Department of Neurology, University of Massachusetts Chan Medical School, Worcester, MA, 01655, USA

[5]Division of Genetics and Cell Biology, IRCCS San Raffaele Scientific Institute, Milano, 20132, Italy

*To whom correspondence should be addressed. Tel: +1 (608) 265-3758; Email: fdilworth@wisc.edu

Present Address: Matthew Man-Kin Wong, Department of Genetics, Stanford University, Palo Alto, CA, 94304, USA

**Supplementary Materials and Methods**

## RT-qPCR

Total RNA from cultured cells was harvested using RNA-STAT60 reagent (Tel-Test # CS-111) according to the manufacturer's protocol. Purified RNA (2000 ng) was subjected to reverse transcriptase reaction in the presence of 2.5 mM dNTP (Thermo Fisher Scientific #10297-018), 30 ng/ml random primers (Thermo Fisher Scientific # 48190011) with 6 U/µl Moloney Murine Leukemia Virus Reverse Transcriptase (New England BioLabs # MO253L). qPCR reactions were performed using PowerUP SYBR Green Master Mix (Thermo Fisher Scientific # A25778) according to the user manual. Expression of genes were normalized to GAPDH expression level using the delta-delta Ct method. *$p<0.05$, **$p<0.01$, ***$p<0.001$, ****$p<0.0001$. Statistical quantification was performed using student's two-tailed t-test on the statistical graphing software GraphPad Prism v6.0.

| **Primer name** | **Sequence** |
|---|---|
| SMCHD1-for | CGACAGATTGTCCAGTTCCTC |
| SMCHD1-rev | CCAATGGCCTCTTCTCTCTG |
| GAPDH-for | TCAAGAAGGTGGTGAAGCAGG |
| GAPDH-rev | ACCAGGAAATGAGCTTGACAAA |
| LAP2-for | CGGACTTCTCCAGTGACGA |
| LAP2-rev | GGACCAGGATTCACTCCGTA |
| CCNA2-for | CTCTACACAGTCACGGGACAAAG |
| CCNA2-rev | CTGTGGTGCTTTGAGGTAGGTC |
| CCNB1-for | GACCTGTGTCAGGCTTTCTCTG |
| CCNB1-rev | GGTATTTTGGTCTGACTGCTTGC |
| CCNB2-for | CAACCAGAGCAGCACAAGTAGC |
| CCNB2-rev | GGAGCCAACTTTTCCATCTGTAC |
| CDK1-for | GGAAACCAGGAAGCCTAGCATC |
| CDK1-rev | GGATGATTCAGTGCCATTTTGCC |
| CDKN3-for | ATGGAGGGACTCCTGACATAGC |
| CDKN3-rev | TCTCCCAAGTCCTCCATAGCAG |
| CENPA-for | GGCGGAGACAAGGTTGGCTAAA |
| CENPA-rev | GGCTTGCCAATTGAAGTCCACAC |
| CENPI-for | GCCTTTGTTGTCGGTCACAGTTC |
| CENPI-rev | AGAAGAGCTGCGCTAGATGGTC |
| CENPE-for | GGAGAAAGATGACCTACAGAGGC |
| CENPE-rev | AGTTCCTCTTCAGTTTCCAGGTG |
| HIST1H3A-for | TCCGCCGTTATCAGAAGTCCAC |
| HIST1H3A-rev | GCTCTGGAAACGCAGGTCTGTT |
| HIST1H2AB-for | GGCGGTGCTTGAGTACCTGAC |
| HIST1H2AB-rev | AAGCTCCTCGTCATTGCGGATG |
| HIST1H1B-for | CCGAAAAAGGCAACCAAGAGTCC |
| HIST1H1B-rev | GTTTTCACACGCCAGCTTCCTAC |
| HIST1H3B-for | GCGAGAAATCGCCCAAGACTTC |
| HIST1H3B-rev | CAAAGAGCCCTACCAAGTAGGC |

## Immunoblotting

Cells were lysed in RIPA buffer (150 mM NaCl, 1% NP-40, 0.5% sodium deoxycholate, 0.1% SDS. 50 mM Tris, pH 8.0) with 1x EDTA-free Protease Inhibitor Cocktail (Roche # 1187358000), left on ice for 5 minutes followed by centrifugation

at 16,000 x g for 10 minutes at 4°C. Supernatants were collected and proteins were quantified using protein assay dye reagent (Biorad #5000006). Western blotting was performed as previously described (1), with 1:1000 SMCHD1 antibody (Bethyl Laboratories #A302-871A) and 1:000 α-Tubulin antibody (Cell Signaling Technology #3873) used in the primary antibody incubation step.

## Supplementary Figures

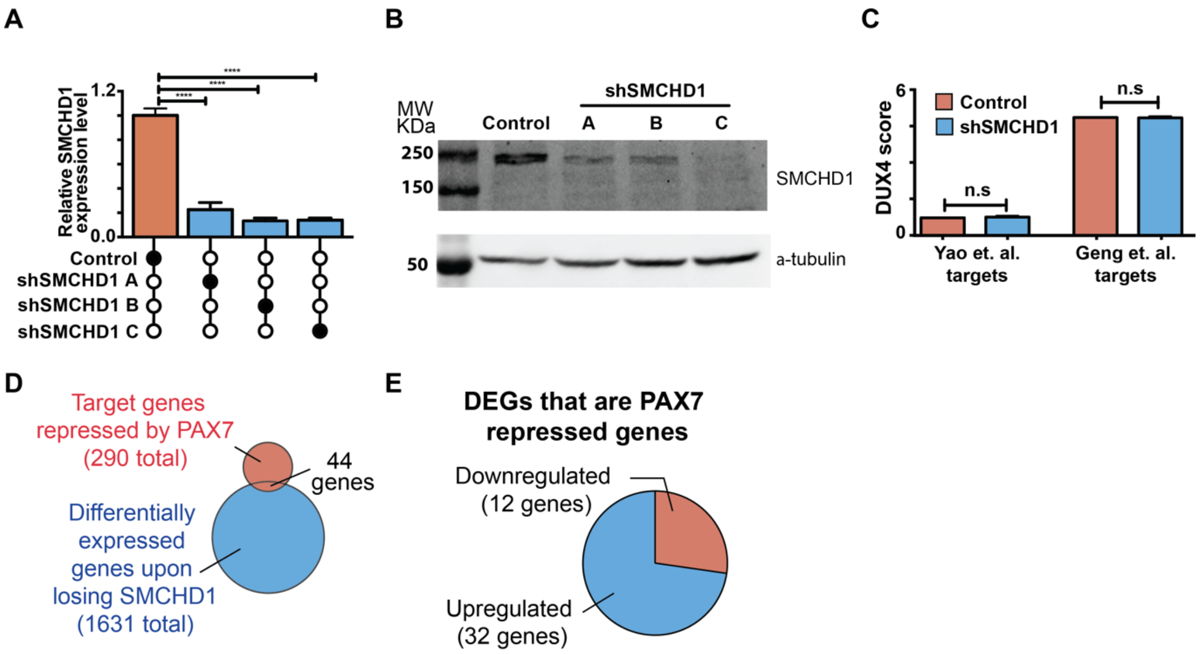


**Supplementary Figure S1** - **(A)** Quantitative PCR results showing the relative expression of *SMCHD1* in myoblasts 5 days after transduction of lentiviral vectors expressing non-silencing shRNA (Control) or shRNA targeting *SMCHD1* (shSMCHD1 A, shSMCHD1 B and shSMCHD1 C). Error bars represent standard deviations of 3 independent experiments. **(B)** (Upper panel) Western blot showing the expression of SMCHD1 and (lower panel) $\alpha$-tubulin of the same blot 5 days after lentiviral transduction. **(C)** DUX4 scores of samples transduced with non-silencing scrambled shRNA (Control) or *SMCHD1* targeting shRNA (shSMCHD1), based on DUX4 targets identified by Yao et. al. (2) and Geng et. al. (3). **(D)** Venn diagram showing the target genes repressed by PAX7 and differentially expressed upon SMCHD1 depletion. **(E)** Number of genes repressed by PAX7 that were down-regulated (red) and up-regulated (blue) upon SMCHD1 depletion.

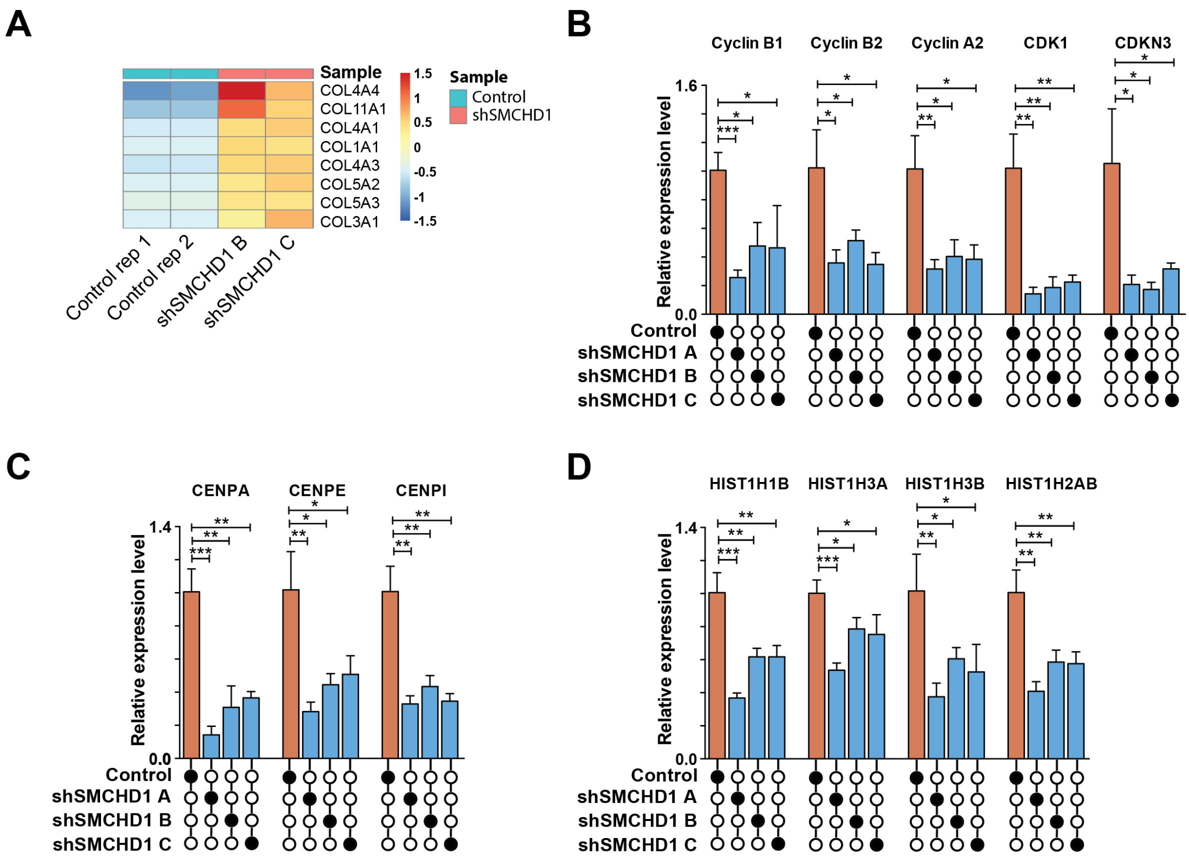


**Supplementary Figure S2** - **(A)** Heatmaps showing the changes in expression of genes encoding collagen subtypes upon losing SMCHD1. **(B-D)** Quantitative PCR results showing the relative expression of mRNA code for (B) cyclins and cyclin dependent kinases, (C) centromere proteins and (D) core histones 5 days after transduction of lentiviral vectors expressing non-silencing scrambled shRNA (Control) or shRNA targeting SMCHD1 (shSMCHD1 A, shSMCHD1 B and shSMCHD1 C). Error bars represent standard deviations of 3 independent experiments.

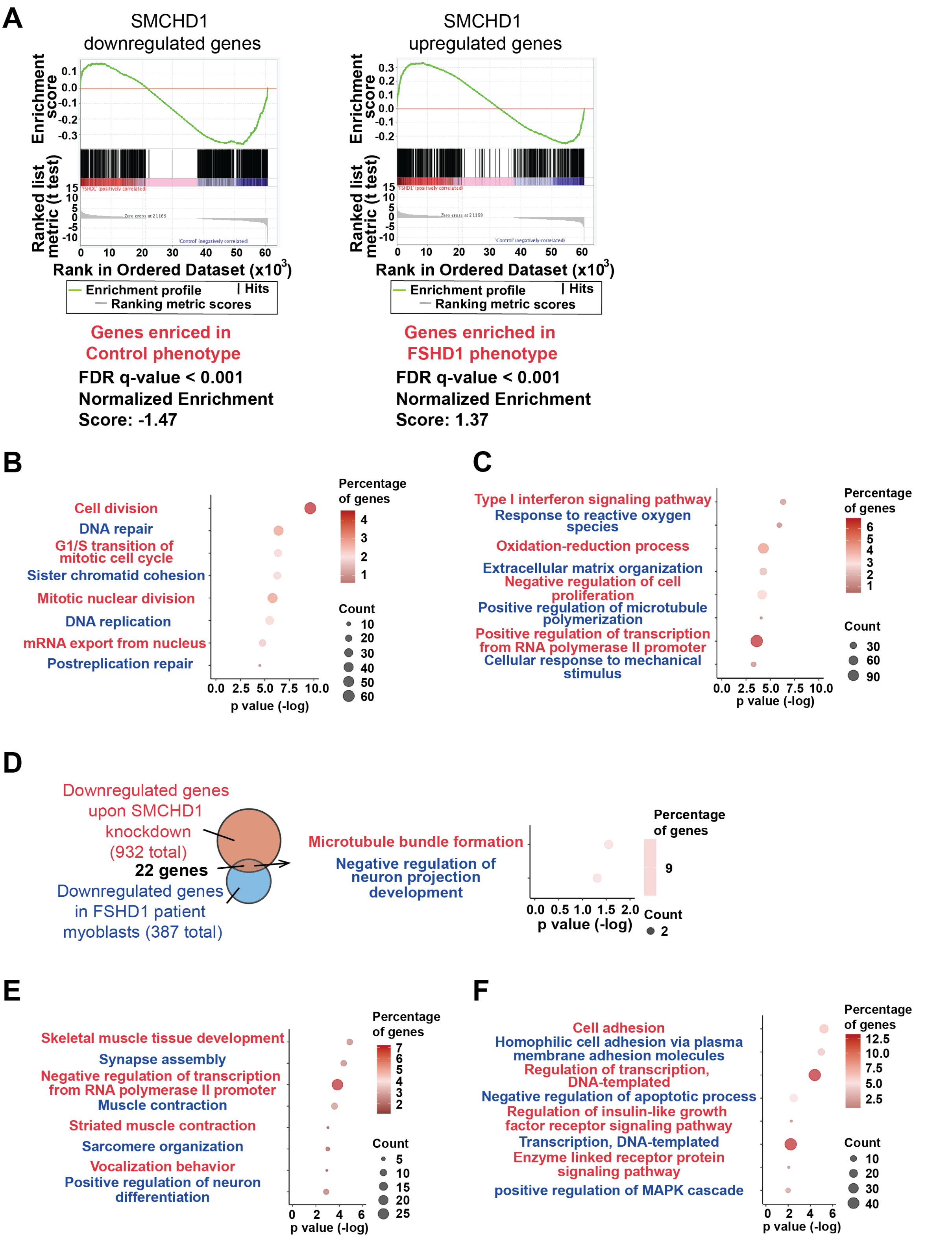


**Supplementary Figure S3 - (A)** Gene set enrichment analysis (GSEA) of DEGs in FSHD1 myoblasts compared to down-regulated (left panel) or up-regulated (right panel) genes upon SMCHD1 depletion. **(B-C)** Dot plots showing the top GO terms of (B) down-regulated and (C) up-regulated genes in primary FSHD2 myoblasts (differentiation day 0) RNA-Seq dataset (GEO accession number GSE143493). **(D)** (Left panel) Venn diagram of commonly down-regulated genes and (Right panel) dot plot of top GO terms of the genes commonly down-regulated in SMCHD1-depleted myoblasts and in FSHD1 myoblasts. **(E-F)** Dot plots showing the top GO terms of (E) down-regulated and (F) up-regulated genes in FSHD1 myoblasts clones having shortened D4Z4 locus (GEO accession number GSE102812).

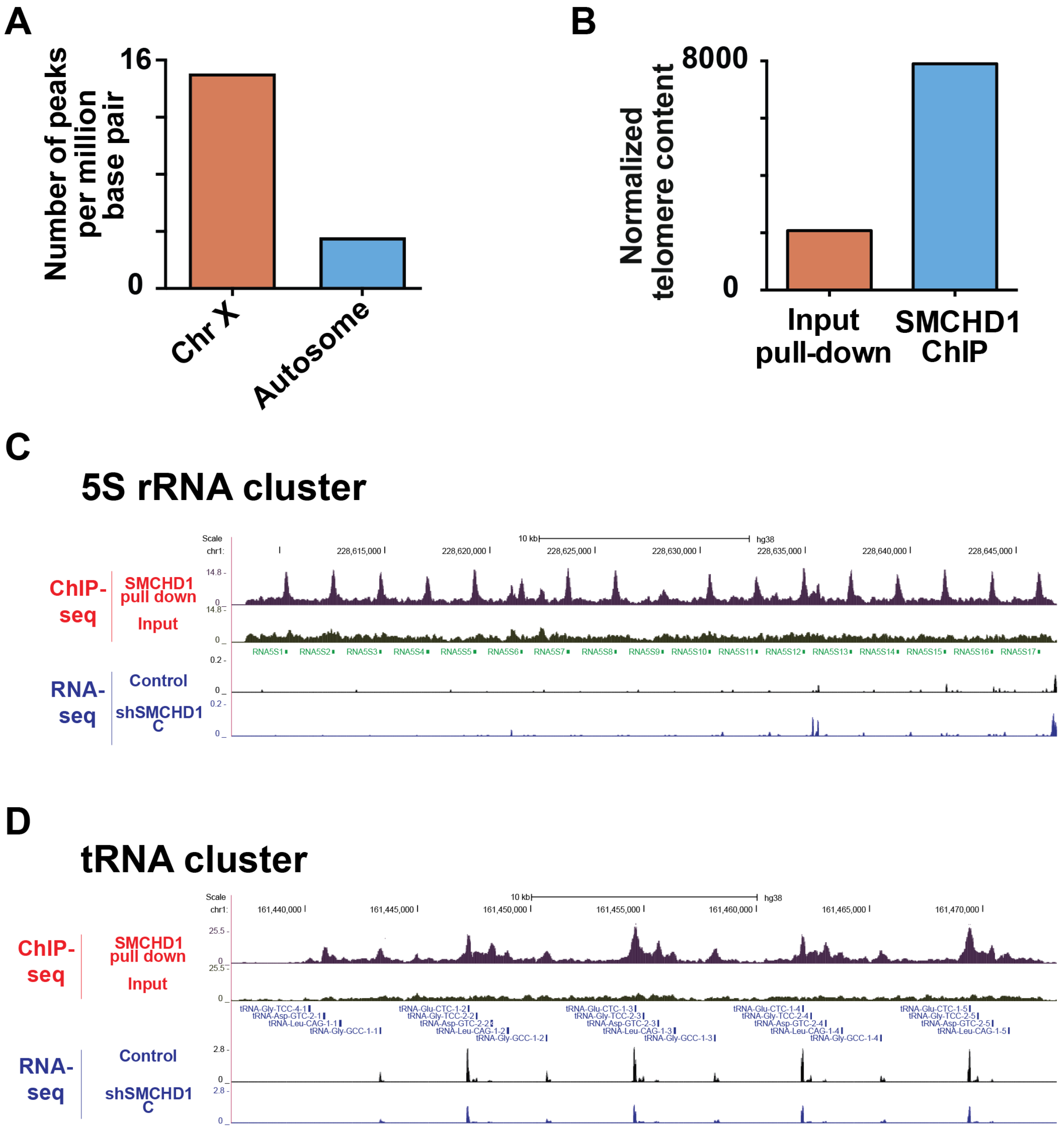


**Supplementary Figure S4 - (A)** Average number of SMCHD1 peaks on autosomes and on chromosome X. **(B)** Telomere content in the input and SMCHD1 pull-down sample calculated by the TelomereHunter software. **(C-D)** UCSC genome browser tracks showing SMCHD1 ChIP-Seq reads and SMCHD1 depletion RNA-Seq reads (control: proliferating myoblasts expressing non-silencing scrambled shRNA, shSMCHD1: proliferating myoblasts expressing shRNA targeting *SMCHD1*) near (C) 5S rRNA cluster, (D) tRNA cluster.

## A

### Enriched *de novo* motif in peaks associated with down-regulated direct targets

| Rank | Motif | p value | Percentage of taget sequence with motif | Percentage of GC normalized scramble sequence with motif | Best match |
|---|---|---|---|---|---|
| 1 | 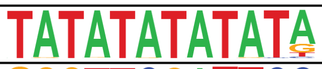 | 1e-50 | 16.82% | 0.03% | SeqBias: TA-repeat |
| 2 | 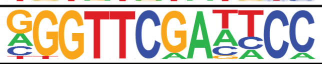 | 1e-41 | 15.40% | 0.21% | OPI1 |
| 3 | 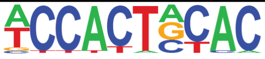 | 1e-36 | 13.74% | 0.22% | PB0091.1_Zbtb3_1 |
| 4 | 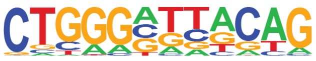 | 1e-34 | 29.15% | 6.04% | ZNF416(Zf) |
| 5 | 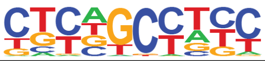 | 1e-33 | 26.30% | 4.69% | ZNF460 |
| 6 | 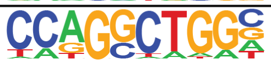 | 1e-32 | 14.93% | 0.78% | SWI5 |

## B

### Enriched *de novo* motif in peaks associated with up-regulated direct targets

| Rank | Motif | p value | Percentage of taget sequence with motif | Percentage of GC normalized scramble sequence with motif | Best match |
|---|---|---|---|---|---|
| 1 | 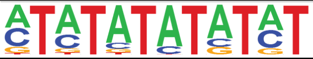 | 1e-57 | 17.94% | 0.02% | SeqBias: TA-repeat |
| 2 | 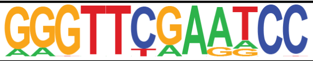 | 1e-47 | 16.37% | 0.22% | OPI1 |
| 3 | 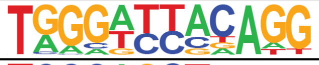 | 1e-28 | 10.31% | 0.18% | PTF1(CTP) |
| 4 | 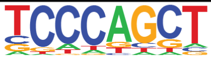 | 1e-27 | 47.53% | 20.79% | ZNF416(Zf) |
| 5 | 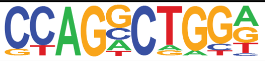 | 1e-27 | 12.56% | 0.81% | Smad2(MAD) |
| 6 | 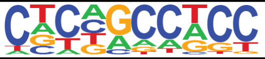 | 1e-26 | 20.63% | 3.88% | ZNF460 |

## C

### Enriched known motif in peaks associated with direct targets

| Motif | Name | p-value (Target VS non-target) | q-value (Target VS non-target) | % of target sequence with motif | % of non-target sequence with motif | % of GC normalized scramble sequence with motif |
|---|---|---|---|---|---|---|
| 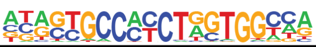 | CTCF | 1e-4 | 0.0108 | 7.35% | 4.07% | 0.36% |

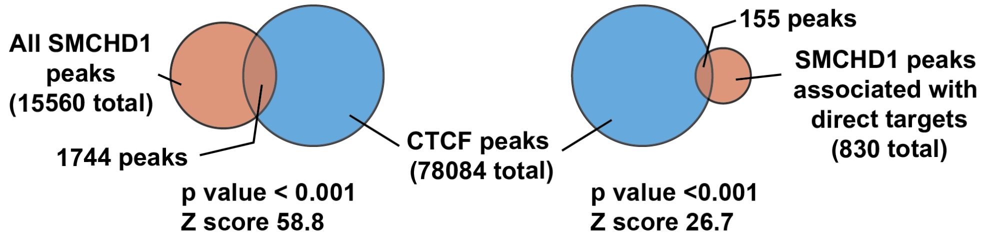


**Supplementary Figure S5 -** HOMER analysis showing the *de novo* search of enriched motifs in SMCHD1 peaks associated with **(A)** down-regulated direct targets and **(B)** up-regulated direct targets upon SMCHD1 depletion. **(C)** (Upper panel) CTCF motif was enriched among the SMCHD1 peaks associated with direct targets. (Lower panel) Number of CTCF peaks colocalized with SMCHD1 peaks. p value and Z score were calculated by permutation test using regioneR.

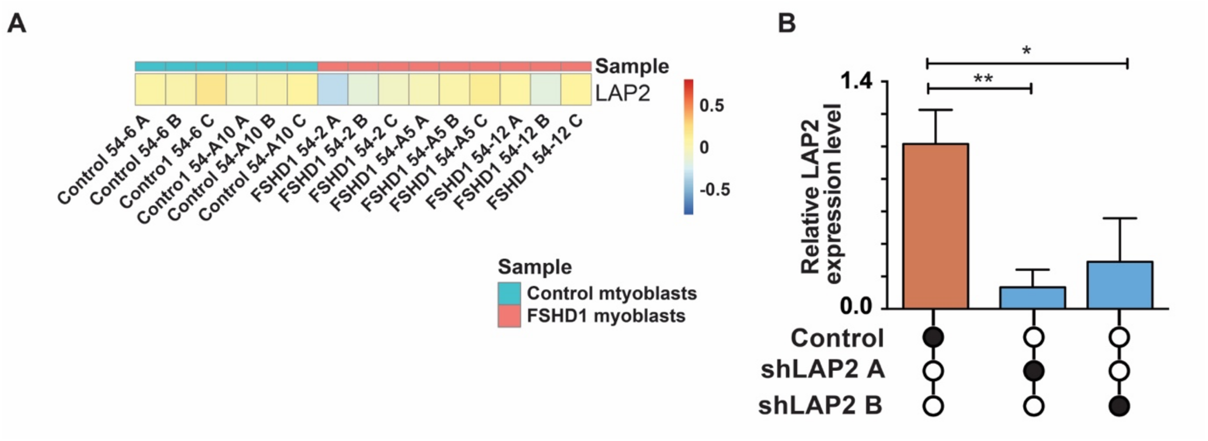


**Supplementary Figure S6**

**(A)** Heatmap showing the expression of LAP2 from control non-FSHD and FSHD1 myoblasts (GEO assession number GSE102812). **(B)** Quantitative PCR results showing the relative expression of *LAP2* after transduction of lentiviral vectors expressing non-silencing shRNA (Control) or shRNA targeting *LAP2* (shLAP2 A and shLAP2 B). Error bars represent standard deviation from 3 independent experiments.

**Supplementary Table S1. List of reagents**

| Name of Regent | company name | Catalog # |
|---|---|---|
| α-Tubulin antibody | Cell Signaling Technology | 3873 |
| BGS | HyClone | SH30541.03 |
| DAPI | Sigma | D9542 |
| Dexamethasone | Sigma | D2915 |
| DMEM | HyClone | SH3024301 |
| dNTP | Thermo Fisher Scientific | 10297-018 |
| EDTA | Fisher Scientific | S311-500 |
| EDTA-free Protease Inhibitor Cocktail | Roche | 1187358000 |
| EdU Cell Proliferation Kit for Imaging | Thermo Fisher Scientific | C10340 |
| FGF | Stemcell Technologies | 78003.2 |
| Formaldehyde | Fisher Scientific | BPBP53125 |
| Gelatin | Sigma | G9391 |
| Glycerol | Fisher Scientific | G33-1 |
| Goat anti-rabbit IgG antibody conjugated to Alexa Fluor 488 | Thermo Fisher Scientific | A-11034 |
| HEPES | Sigma | H3784 |
| HGF | Stemcell Technologies | 78019.2 |
| IRDye 680RD donkey anti-Rabbit IgG antibody | Li-Cor | LIC-926-68073 |
| IRDye 800CW goat anti-Mouse IgG antibody | Li-Cor | LIC-926-32210 |
| KAPA HyperPrep Kit | Roche | 07962363001 |
| KAPA stranded RNA-seq kit | Roche | 07962169001 |
| M-MuLV Reverse Transcriptase | New England BioLabs | MO253L |
| Medium 199 | Thermo Fisher Scientific | 31100035 |
| Na-deoxycholate | Sigma | D6750 |
| NaCl | Sigma | S9888 |
| Normal rabbit IgG | Thermo Fisher Scientific | 10500C |
| NP-40 | Sigma | 74385 |
| PBS | Gibco | 21600-069 |
| pDONR223-LAP2β plasmid | Genome editing and molecular biology facility of the University of Ottawa | N/A |
| Penicillin/streptomycin | Wisent Bioproducts | 450-201-EL |
| pLenti-EF1a-Blank vector | Applied Biological Materials | LV588 |
| pMD2.G vector | Addgene | 12259 |
| Polybrene | Sigma | H9268 |

| | | |
|---|---|---|
| Polyethylenimine | Polysciences | 23966 |
| PowerUP SYBR Green Master Mix | Thermo Fisher Scientific | A25778 |
| Protein Assay Dye Reagent | Bio-Rad | 5000006 |
| psPAX2 vector | Addgene | 12260 |
| PureLink RNA mini kit | Thermo Fisher Scientific | 12183018A |
| Random Primers | Thermo Fisher Scientific | 48190011 |
| Ribo-Zero Magnetic Gold Kit | Illumina | MRZG12324 |
| RNA-STAT60 reagent | Tel-Test | CS-111 |
| SDS | Sigma | L3771 |
| SMCHD1 antibody (for ChIP-Seq) | Abcam | ab31865 |
| SMCHD1 antibody (for western blot) | Bethyl Laboratories | A302-871A |
| Sucrose | Fisher Scientific | S6500 |
| Tris base | Fisher Scientific | BP154-1 |
| Triton X-100 | Sigma | T8787 |
| Vitamin B12 | Sigma | V2876 |
| Zinc sulfate | Fisher Scientific | Z68500 |

**Supplementary Table S2. Software and databases used in data analysis**

| Software and algorithms | Reference | Web Site |
|---|---|---|
| BEDTools | (4) | https://bedtools.readthedocs.io/en/latest/ |
| BETA algorithm | (5) | http://cistrome.org/BETA/ |
| bowtie2 (v2.3.5.1) | (6) | http://bowtie-bio.sourceforge.net/bowtie2/index.shtml |
| DAVID (v6.8) | (7-8) | https://david.ncifcrf.gov |
| DESeq2 (v1.30.0) | (9) | https://bioconductor.org/packages/release/ bioc/html/DESeq2.html |
| EnhancedVolcano (v1.6.0) | | https://bioconductor.org/packages/release/ bioc/html/EnhancedVolcano.html |
| EnrichedHeatmap (v1.18.2) | (10) | https://bioconductor.org/packages/release/ bioc/html/EnrichedHeatmap.html |
| Fiji (v2.1.0) | (11) | https://imagej.net/software/fiji/ |
| ggplot2 (v3.3.0) | (12) | https://ggplot2.tidyverse.org |
| GSEA software | (13-14) | https://www.gsea-msigdb.org/gsea/index.jsp |
| HOMER (v4.11) | (15) | http://homer.ucsd.edu/homer/motif/ |
| MACS2 | (16) | https://github.com/macs3-project/MACS |
| pheatmap (v1.0.12) | | https://cran.r-project.org/web/packages/ pheatmap/index.html |
| GraphPad Prism (v6.0) | | https://www.graphpad.com/scientific-software/prism/ |
| R (v4.0.0) | | https://www.r-project.org |
| regioneR (v 3.14) | (17) | http://bioconductor.org/packages/release/ bioc/html/regioneR.html |
| Rsubread (v1.6.2) | (18) | https://bioconductor.org/packages/release/ bioc/html/Rsubread.html |
| samtools | (19) | http://samtools.sourceforge.net |
| STAR (v2.7.5a) | (20) | https://github.com/alexdobin/STAR |
| TelomereHunter | (21) | https://pypi.org/project/telomerehunter/ |

**Supplementary Table S3. List of shRNA vectors**

The following shRNA vectors were purchased from GeneCopoeia (MD, USA):

| **shRNA Vector** | **Target sequence** |
|---|---|
| psi-LVRU6MH-shControl | GCTTCGCGCCGTAGTCTTA |
| psi-LVRU6MH-shSMCHD1 A | GGACGGTGTACTTGTTTGATC |
| psi-LVRU6MH-shSMCHD1 B | GGGATTATCCGTTATCATCCA |
| psi-LVRU6MH-shSMCHD1 C | CCTATTGGTGCGTTAAGAATT |
| psi-LVRU6GP-shControl | GCTTCGCGCCGTAGTCTTA |
| psi-LVRU6GP-shLAP2 A | GGAACAGAATCAAGATCTTCT |
| psi-LVRU6GP shLAP2 B | GCTGAAACTATAATGGCTTCA |

**Reference:**

1. Singh,K., Cassano,M., Planet,E., Sebastian,S., Jang,S. M., Sohi, G., Faralli, H., Choi, J., Youn, H. D., Dilworth, F. J., & Trono, D. (2015). A KAP1 phosphorylation switch controls MyoD function during skeletal muscle differentiation. *Genes Dev*, 29, 513–525.

2. Yao,Z., Snider,L., Balog,J., Lemmers,R.J., Van Der Maarel,S.M., Tawil,R. and Tapscott,S.J. (2014). DUX4-induced gene expression is the major molecular signature in FSHD skeletal muscle. *Hum Mol Genet*, 23, 5342–5352.

3. Geng,L.N., Yao,Z., Snider,L., Fong,A.P., Cech,J.N., Young,J.M., van der Maarel,S.M., Ruzzo,W.L., Gentleman,R.C., Tawil,R., et al. (2012). DUX4 activates germline genes, retroelements, and immune mediators: implications for facioscapulohumeral dystrophy. *Dev Cell*, 22, 38–51.

4. Quinlan,A.R. and Hall,I.M. (2010). BEDTools: a flexible suite of utilities for comparing genomic features. *Bioinformatics*. 26, 841–842.

5. Wang,S., Sun,H., Ma,J., Zang,C., Wang,C., Wang,J., Tang,Q., Meyer,C.A., Zhang,Y. and Liu,X.S. (2013). Target analysis by integration of transcriptome and ChIP-seq data with BETA. *Nat Protoc*. 8, 2502–2515.

6. Langmead,B. and Salzberg,S.L. (2012). Fast gapped-read alignment with Bowtie 2. *Nat Methods*. 9, 357–359.

7. Huang,D., Sherman,B.T. and Lempicki,R.A. (2009). Systematic and integrative analysis of large gene lists using DAVID bioinformatics resources. *Nat Protoc*. 4, 44–57.

8. Huang,D., Sherman,B.T. and Lempicki,R.A. (2009). Bioinformatics enrichment tools: paths toward the comprehensive functional analysis of large gene lists. *Nucleic Acids Res*. 37, 1–13.

9. Love,M.I., Huber,W. and Anders,S. (2014). Moderated estimation of fold change and dispersion for RNA-seq data with DESeq2. *Genome Biol*. 15, 550.

10. Gu,Z., Eils,R., Schlesner,M. and Ishaque,N. (2018). EnrichedHeatmap: an R/Bioconductor package for comprehensive visualization of genomic signal associations. *BMC Genomics*. 19, 234.

11. Schindelin,J., Arganda-Carreras,I., Frise,E., Kaynig,V., Longair,M., Pietzsch,T., Preibisch,S., Rueden,C., Saalfeld,S., Schmid,B., et. al. (2012). Fiji: an open-source platform for biological-image analysis. *Nat Methods*. 9, 676–682.

12. Wickham,H (2016). ggplot2: Elegant Graphics for Data Analysis, Springer-Verlag, New York.

13. Subramanian,A., Tamayo,P., Mootha,V.K., Mukherjee,S., Ebert,B.L., Gillette,M.A., Paulovich,A., Pomeroy,S.L., Golub,T.R., Lander,E.S., et. al. (2005). Gene set enrichment analysis: a knowledge-based approach for interpreting genome-wide expression profiles. *Proc Natl Acad Sci U S A*. 102, 15545–15550.

14. Mootha,V.K., Lindgren,C.M., Eriksson,K.F., Subramanian,A., Sihag,S., Lehar,J., Puigserver,P., Carlsson,E., Ridderstråle,M., Laurila,E., et. al. (2003). PGC-1alpha-responsive genes involved in oxidative phosphorylation are coordinately downregulated in human diabetes. *Nat Genet*. 34, 267–273.

15. Heinz,S., Benner,C., Spann,N., Bertolino,E., Lin,Y.C., Laslo,P., Cheng,J.X., Murre,C., Singh,H. and Glass,C.K. (2010). Simple combinations of lineage-determining transcription factors prime cis-regulatory elements required for macrophage and B cell identities. *Mol Cell*. 38, 576–589.

16. Zhang,Y., Liu,T., Meyer,C.A., Eeckhoute,J., Johnson,D.S., Bernstein,B.E., Nusbaum,C., Myers,R.M., Brown,M., Li,W., et. al. (2008). Model-based analysis of ChIP-Seq (MACS). *Genome Biol*. 9, R137.

17. Gel,B., Díez-Villanueva,A., Serra,E., Buschbeck,M., Peinado,M.A. and Malinverni,R. (2016). regioneR: an R/Bioconductor package for the association analysis of genomic regions based on permutation tests. *Bioinformatics*. 32, 289–291.

18. Liao,Y., Smyth,G.K. and Shi,W. (2019). The R package Rsubread is easier, faster, cheaper and better for alignment and quantification of RNA sequencing reads. *Nucleic Acids Res*. 47, e47.

19. Li,H., Handsaker,B., Wysoker,A., Fennell,T., Ruan,J., Homer,N., Marth,G., Abecasis, G., Durbin,R. and 1000 Genome Project Data Processing Subgroup (2009). The Sequence Alignment/Map format and SAMtools. *Bioinformatics*. 25, 2078–2079.

20. Dobin,A., Davis,C.A., Schlesinger,F., Drenkow,J., Zaleski,C., Jha,S., Batut,P., Chaisson,M. and Gingeras,T.R. (2013). STAR: ultrafast universal RNA-seq aligner. *Bioinformatics*. 29, 15–21.

21. Feuerbach,L., Sieverling,L., Deeg,K.I., Ginsbach,P., Hutter,B., Buchhalter,I., Northcott,P.A., Mughal,S.S., Chudasama,P., Glimm,H., et. al. (2019). TelomereHunter - in silico estimation of telomere content and composition from cancer genomes. *BMC Bioinformatics*. 20, 272.
